# Observations on the Acetite of Lead

**Published:** 1802-06

**Authors:** Michael Ryan

**Affiliations:** Kilkenny


### *Obſervations on the Acetite of Lead; communicated by Mr.* Michael Ryan, *of Kilkenny.*

ALTHOUGH phyſicians are in the daily practice of preſcribing medicines of the moſt deleterious powers with impunity, it is ſingular enough to find one belonging to this claſs, of very inferior agency, rejected as inadmiſſable by the writers on the Materia Medica. Thus arſenic, belladona, digitalis, and many others, are not only taken in modified doſes with ſafety, but with ſingular advantage; while the acetite of lead, in the ſmalleſt quantity, is univerſally dreaded, from the pernicious influence it is ſuppoſed to exert on the living muſcular fibre. Here, a queſtion very naturally preſents itſelf. Is this generally received opinion of its deſtructive power, the reſult of experiment and accurate obſervation? From what I have had an opportunity of witneſſing, I do not think it is.

A phyſician, whoſe writings have eminently contributed to embelliſh as well as to improve the ſcience of medicine, has, by his different Eſſays on this ſubject, given currency to the idea of lead, in any ſhape, being too hoſtile to the human conſtitution to admit of its being employed as a remedy in the cure of diſeaſes. The intelligent reader will readily anticipate the learned writer I allude to.

Sir

Sir George Baker has taken great pains, and with fome appearance of truth, to prove in many inftances the exiftence of cholic from the fpecific operation of lead. The varieties of this difeafe, as Colica Pictonum, Derbyfhire Colic, Morbus Colicus Damnoniorum, &c. are all by him afcribed to the action of this metal in one fhape or other. That fuch is the fact on fome occafions it is not intended here to deny; but whoever reads with attention, and forms an impartial judgment of the numberlefs cafes of colic, which are faid to derive their origin from this fource, before he gives his unqualified affent to them, muft firft confider this poifon more virulent and unmanageable than any other in the vegetable or mineral kingdom, even arfenic itfelf not excepted. The very fmall portion of it, which is faid to be followed with fuch untoward confequences, is above all belief, and will find very little credit in this age of daring experiment and enterprife. Even Sir G. Baker himfelf, by what follows, does not attempt to fay that the evidence in favor of this opinion is demonftrative, though he does not feem to queftion its high degree of probability. Speaking of the grounds on which this belief refts, he fays, "This opinion muft ftill await the judg-"ment of future obfervation and inquiry. On the one hand, "from the nature of the fubject, it is not reducible to the cer-"tainty of demonftration; on the other hand, it does not ap-"pear that its probability has hitherto been leffened either by "reafoning or experiment." Vide London Med. Trans. vol. iii. p. 408. In this ftate of uncertainty the only method of adjufting the point in queftion (and furely it is an object of the firft moment) is by adminiftering certain preparations of lead in the fame guarded and cautious manner as is practifed with refpect to other medicines of fingularly active powers. What has immediately in this way come within the fphere of my obfervation, I fhall relate with candour, and leave the reader to judge how far the practice may be imitated with fafety or advantage.

About fix years ago a very obftinate cafe of fpitting of blood being committed to my care, every remedy ufually reforted to on fuch occafions was made ufe of to endeavour to ftop the difcharge, but to no purpofe. Happening to recollect fome obfervations made by Dr. Reynolds in the third volume of the L. Medical Tranfactions with refpect to the ufe of acetite of lead in hæmoptyfis, I determined to adopt this practice in the prefent inftance; but as the danger was very imminent, the medicine was given in the dofe of two grains every third hour inftead of one grain every fixth hour, as prefcribed by Dr. Reynolds. Before the fourth dofe was adminiftered, the bleeding from the lungs began to ceafe, and very fhortly after totally difappeared. The medicine was continued for feveral days without without the patient experiencing any pain in the bowels, or any other difagreeable confequence whatfoever.

Encouraged by the fortunate event of this cafe, I never let flip an opportunity of giving the acetite of lead in every dangerous inftance of hæmoptyfis that came under my direction. In fact, two patients of mine have been taking it for upwards of a week, very lately, in the dofe of fix grains every fixth hour; and though they were minutely queftioned as to its effects on the bowels, they never complained of pain, griping, or any other unpleafant fenfation; nay, contrary to the generally received opinion, no coftivenefs or conftipation was induced that could not be immediately removed by a moderate dofe of caftor oil or any other mild aperient medicine.

On looking over my Book of Reports, I find, befides the two juft now mentioned, a detail of fix cafes, in which not lefs than twenty or thirty grains of acetite of lead were given daily for eight or ten days fucceffively with perfect fafety, and no precaution was taken to obviate its poifonous quality, except by adding three or four drops of tinct. opii to each bolus or draught, which ever happened to be prefcribed. If thefe be facts, whofe authenticity cannot be called in queftion, (and I do not think I could be deceived) one medicine more will be reftored to the Materia Medica, which promifes incalculable advantage to the healing art, from which the timidity of phyficians had completely banifhed it.*

* The Editors inform Mr. R. that fince the appearance of Dr. Reynolds's paper, the acetite of lead has been ufed very freely in London.

